# The decreased mean platelet volume is associated with poor prognosis in patients with oropharyngeal cancer treated with radiotherapy

**DOI:** 10.1186/s13014-020-01702-4

**Published:** 2020-11-07

**Authors:** Daniela Delago, Olivia Knittelfelder, Gabriele Jakse, Katarzyna Lukasiak, Sabine Reinisch, Wilfried Renner, Heidi Stranzl-Lawatsch, Richard Partl, Tanja Langsenlehner

**Affiliations:** 1 Department of Therapeutic Radiology and Oncology, Comprehensive Cancer Center, Medical University of Graz, Auenbruggerplatz 32, 8036 Graz, Austria; 2 Department of Otorhinolaryngology, Comprehensive Cancer Center, Medical University of Graz, Auenbruggerplatz 26, 8036 Graz, Austria; 3 Clinical Institute of Medical and Chemical Laboratory Diagnostics, Medical University of Graz, Auenbruggerplatz 15, 8036 Graz, Austria

**Keywords:** Oropharyngeal cancer, Biomarker, Mean platelet volume, Prognostic factor, Outcome

## Abstract

**Background:**

There is considerable evidence that platelets contribute to cancer growth and metastatic dissemination. In recent studies, altered mean platelet volume (MPV) has been associated with prognosis in different types of cancer. However, the prognostic role of the MPV in head and neck squamous cell cancer (HNSCC) is currently discussed controversially. The present study was performed to analyze and further elucidate the prognostic significance of the MPV in HNSCC.

**Methods:**

A total of 319 oropharyngeal squamous cell cancer (OPSCC) patients treated with radiotherapy at a tertiary academic center were enrolled in the present study. Kaplan–Meier method as well as uni- and multivariate Cox proportional hazards were used to evaluate the impact of MPV on cancer-specific survival (CSS), locoregional control (LC) and recurrence-free survival (RFS).

**Results:**

The median MPV was 10.30 fL (mean 10.26 ± 1.17fL). Univariate analyses showed a significant association of the MPV with CSS (HR 0.85, 95% CI 0.74–0.98, *p* = 0.025), LC (HR 0.86, 95% CI 0.74–0.99, *p* = 0.034) and RFS (HR 0.87, 95% CI 0.76–0.996; *p* = 0.043). In multivariate analysis, the MPV remained an independent prognostic factor for CSS (HR 0.77, 95% CI 0.63–0.93, *p* = 0.008), LC (HR 0.80, 95% CI 0.65–0.98, *p* = 0.030), and RFS (HR 0.83, 95% CI 0.685–0.999, *p* = 0.049).

**Conclusions:**

Our findings indicate that the MPV is a prognostic marker in OPSCC patients and may contribute to future individual risk assessment.

## Background

Oropharyngeal squamous cell carcinoma (OPSCC) is a common malignancy of the head and neck and includes cancers of the tonsils, base of the tongue, soft palate, and upper lateral and posterior pharyngeal walls. Worldwide age-adjusted incidence rates for men and women are 3.8 and 0.8 per 100 000 populations respectively, with a substantial variation in different regions and countries [1, 2]

Despite several advances in the management of OPSCC including novel imaging as well as innovations in surgical and radiotherapeutic techniques, the overall survival of patients with OPSCC still remains around 50% at 5 years, primarily because of locoregional and/or systemic recurrence of disease [2]. In recent years, many efforts have been undertaken to identify novel molecular and cellular prognostic biomarkers in order to improve the prediction of the risk of OPSCC recurrence, however, high costs of analyses, time-consuming preparation and lack of standardization limit their application in routine clinical practice [3]. In addition, there is increasing data demonstrating that radiomics provides important prognostic information for the risk assessment of specific outcomes in head and neck cancer [4, 5]. The underlying idea of radiomics is that the molecular and cellular high risk features could translate into heterogeneous tumor metabolism and anatomy. Thus, the integration of quantitative imaging information with genomics, transcriptomics, proteomics, metabolomics could efficiently unravel biological mechanisms, however, further analyses of the underlying biological processes are needed.

Platelets play a major role in cancer progression and metastasis. There is emerging evidence suggesting that activated platelets mediate tumor progression by interacting with various cell types and participating in tumor proliferation related processes [6]. In addition, platelets have been shown to promote cancer angiogenesis by releasing angiogenic growth factors such as vascular endothelial growth factor (VEGF) [7].

 Platelet count is determined by the balance between the rate of production and consumption of platelets. A highly pro-inflammatory cancer phenotype may lead to enhanced megakaryopoiesis and increased platelet production triggered by tumor-related cytokines, however, platelet consumption caused by hypercoagulation may cause a normal platelet count. A normal platelet count could therefore conceal the presence of a highly hypercoagulative and pro-inflammatory cancer phenotype in the presence of efficient compensatory mechanisms [8].

Mean platelet volume (MPV), the most commonly used measure of platelet size, represents a surrogate marker of platelet activation [9]. Large platelets have been suggested to be more reactive and more likely to aggregate which leads to their faster consumption. The observation of decreased platelet size in cancer patients has been explained by an increased cancer-associated platelet activation and exhaustion [9, 10]. In this context, a low MPV may reflect degranulated “exhausted” platelets that have already secreted their potentially tumor growth-promoting cytokines, and thus are associated with a worse outcome in cancer patients [11]. In addition, an increased release of small platelets from the bone marrow may be the result of enhanced megakaryopoiesis triggered by tumor-related cytokines.

Altered MPV levels have previously been analyzed as a prognostic and predictive biomarker in cancer patients and have been associated with prognosis in several cancer entities such as gastric, bladder, renal, endometrial, non-small cell lung cancer, and hepatocellular carcinoma [12–18]. However, there is currently limited knowledge about the impact of the MPV on outcome in patients with head and neck cancer. Jank et al. analyzed the prognostic value of perioperative platelet indices in 122 head and neck squamous cell cancer (HNSCC) patients treated with surgery and postoperative radiotherapy and concluded that the pre-treatment MPV is not a useful biomarker in HNSCC patients [19]. Park et al. evaluated the prognostic role of the combination of a platelet (COP)-MPV score in 40 oral cancer patients and detected a significant association between the COP-MPV score and survival [20]. In contrast, an additional study including 115 HNSCC patients did not reveal a significant relationship between the COP-MPV score and prognosis [21].

The aim of the present study was to evaluate and further clarify the prognostic value of the pre-treatment MPV for cancer-specific survival (CSS), locoregional control (LC) and recurrence-free survival in a cohort of 319 European OPSCC cancer patients treated with definitive or postoperative radio (chemo-) therapy.

## Materials and methods

The study population comprised 319 patients with primary OPSCC who were treated at a tertiary academic center from 01/2002 to 10/2017. All patients enrolled in this study satisfied the following eligibility criteria: (a) histologically confirmed primary squamous cell carcinoma; (b) no evidence of distant metastasis; and (c) no evidence of other malignancies.

A total of 179 patients (56.1%) underwent definitive radio (chemo-) therapy. Definitive radio (chemo-)therapy was combined with docetaxel, cisplatin, 5-fluorouracil (TPF)-based induction chemotherapy in 57 patients. A total of 152 patients (84.9%) received concurrent chemotherapy, mostly consisting of a platinum-based regimen, although targeted therapy such as cetuximab was also used. A total of 140 patients (43.9%) were treated with surgery and postoperative RT, among them, 80 received concomitant chemotherapy.

All patients underwent radiotherapy with 6 MV photon linear accelerators. The dose-fractionation regimen was either standard fractionation or a simultaneous integrated boost (SIB) protocol. Standard fractionation radiotherapy was delivered up to a total dose of 70.0 Gy in 35 fractions (2.0 Gy per fraction/5 × per week). The prescription dose to primary lesions or positive nodes ranged from 66 to 70 Gy, prophylactic nodal areas were irradiated at doses of 50 Gy. The SIB radiation schedules consisted of 5 × 2 Gy or 5 × 2.2 Gy /week to 70 Gy or 70.4 Gy to clinically manifest sites of gross disease and 5 × 1.6 Gy or 1.69 Gy/week to 56 Gy or 54 Gy to adjacent lymphatic drainage regions at risk for subclinical metastasis.

The radiation method was either three-dimensional conformal radiotherapy or intensity modulated radiotherapy (IMRT), including volumetric modulated arc therapy (VMAT). Patients treated with postoperative radiotherapy received standard fractionation RT up to a total dose of 60–70 Gy at 2 Gy per fraction, depending on risk factors such as resection margin and tumor stage.

The MPV, defined as the average size of platelets found in the blood of patients, was measured in treatment- naïve OPSCC patients prior to the initiation of tumor-specific therapy using standard clinical testing methodology (reference level 7–13 fL). Clinical staging was performed according to the 7th edition of American Joint Committee on Cancer (AJCC) staging in oropharyngeal cancer.

Clinical follow-up was conducted both at the Department of Therapeutic Radiology and Oncology and at the Department of Otorhinolaryngology according to institutional guidelines. Complete physical examination was performed every 3 months (years 1–2)/every 6 months (years 3–5), and annually thereafter, whereas imaging was performed as indicated by clinical examination.

The study complied with the Declaration of Helsinki and was performed according to the national law. The protocol has been approved by the local Ethical Committee (approval number: EK 29-273 ex 16/17). As this is a retrospective nonintervention study, the institutional review board waived the need for written informed consent from the participants.

### Statistical analysis

In order to analyze the prognostic role of the MPV for cancer-specific outcome, the study endpoints evaluated in the present study included CSS, LC, and PFS that directly reflect cancer prognosis but not overall survival that may be influenced by several other conditions or diseases. The primary endpoint was CSS defined as the time from OPSCC diagnosis to the date of OPSCC cancer-related death. The secondary endpoints included LC, defined as no evidence of recurrence or progression of the primary tumor and neck lymph nodes, and RFS, defined as the time from the first day of treatment to the date of the development of locoregional recurrence and/or distant metastases, respectively.

The relationship between the MPV and other clinico-pathological features was studied by non-parametric tests. Cox proportional hazards analysis was performed to calculate the hazard ratio (HR) and 95% confidence interval (CI) to evaluate the influence of the MPV on the clinical endpoints. Multivariate Cox proportional analysis was performed to determine the influence of potential confounders and included variables significantly associated with CSS, LC and RFS in univariate analysis. MPV was further dichotomized into a binary variable with an empiric cut-off at the 25th percentile of its distribution. Patients’ clinical end points were calculated using the Kaplan–Meier method and compared by the log-rank test. Median follow-up was estimated with a reverse Kaplan–Meier estimator according to Schemper and Smith [22].

All statistical analyses were performed using the Statistical Package for Social Sciences version 25.0.0 (SPSS Inc., Chicago, IL, USA). A two-sided *p* < 0.05 was considered statistically significant.

## Results

A total of 319 oropharyngeal cancer patients were included in the present analysis. The median age at time of diagnosis was 59 years (mean 58.7 ± 10 years). The median pre-treatment MPV was 10.26 fL (mean 10.3 ± 1.17 fL), the median platelet count was 254.5 G/L (mean 269.85 ± 88.69 G/L), respectively. Baseline patient and treatment characteristics as well as the correlation between the MPV and baseline characteristics are displayed in Table 1. The MPV significantly correlated with alcohol consumption (*p* = 0.009), no significant associations were found between the MPV and the remaining clinico-pathological parameters (all *p* > 0.05).

**Table 1:** Patient characteristics and correlation between the mean platelet volume and patient and treatment characteristics

Parameter	N (%)	MPV, median (mean ± SD)	*p* value
Sex			
Male	244 (76.5%)	10.30 (10.23 ± 1.17)	0.618
Female	75 (23.5%)	10.20 (10.35 ± 1.15)	
Age at diagnosis			
< 60	168 (52.7)	10.20 (10.22 ± 1.27)	0.432
> 60	151 (47.3%)	10.30 (10.30 ± 1.04)	
Smoking status			
Former or never	121 (37.9%)	10.30 (10.22 ± 1.18)	0.673
Current	191 (59.9%)	10.30 (10.27 ± 1.16)	
Missing data	7 (2.2%)		
Alcohol consumption			
Former or never	189 (59.2%)	10.40 (10.37 ± 1.13)	0.009
Current	114 (35.7%)	10.10 (10.03 ± 1.18)	
Missing data	16 (5%)		
HPV status			
Negative	23 (7.2%)	10.00 (9.94 ± 1.15)	0.110
Positive	37 (11.6%)	10.50 (10.43 ± 0.89)	
Missing data	259 (81.2%)		
Tumor grade			
G 1/2	135 (42.3%)	10.20(10.24 ± 1.12)	0.270
G 3/4	180 (56.4%)	10.40 (10.28 ± 1.21)	
Missing data	4 (1.3%)		
Tumor stage			
T 1/2	116 (36.4%)	10.30 (10.24 ± 1.29)	0.518
T 3/4	197 (61.8%)	10.20 (10.24 ± 1.20)	
Missing data	6 (1.9%)		
Nodal involvement			
N0	34 (10.7%)	10.40 (10.35 ± 1.06)	0.694
N+	281 (88.1%)	10.30 (10.25 ± 1.18)	
Missing data	4 (1.3%)		
Surgery			
Yes	140 (43.9%)	10.40 (10.28 ± 1.27)	0.275
No	179 (56.1%)	10.20(10.25 ± 1.09)	
Induction chemotherapy			
Yes	63 (19.7%)	10.30 (10.27 ± 0.95)	0.882
No	256 (80.3%)	10.30 (10.26 ± 1.22)	
Concomitant chemotherapy			
Yes	87 (27.3%)	10.05 (9.97 ± 1.61)	0.141
No	232 (72.7%)	10.30 (10.37 ± 0.94)	
Chemo-/immunotherapy			
No	86 (26.9%)	10.05 (9.97 ± 1.61)	0.338
Erbitux	38 (11.9%)	10.35 (10.38 ± 1.05)	
Cisplatin	194 (60.8%)	10.30 (10.37 ± 0.92)	
RTx technique			
3D conformal	144 (45.1%)	10.30 (10.24 ± 1.37)	0.684
IMRT	175 (54.9%)	10.20 (10.28 ± 0.97)	
RTx total dose (Gy)			
≤ 60	61 (19.1%)	10.2 (10.35 ± 1.05)	0.779
> 60 to < 70	75 (23.5%)	10.40 (10.20 ± 1.42)	
≥ 70	183 (57.4%)	10.30 (10.26 ± 1.09)	

n, number of patients; SD, standard deviation; MPV, mean platelet volume; IMRT, intensity modulated radiation therapy

Median follow-up time was 66 months (95% CI 60.5 to 71.5 months). During this period, 89 patients (27.9%) developed disease recurrence, in 78 patients (24.5%), locoregional failure was detected. A total of 70 patients (21.9%) died from OPSCC.

In univariate analysis, the MPV was significantly associated with CSS (HR 0.85, 95% CI 0.74–0.98, *p* = 0.025). Furthermore, univariate analysis identified smoking status, alcohol consumption, tumor stage, surgical resection, induction chemotherapy, and total platelet count as significant prognostic factors for CSS (Table 2). In a subsequent multivariate analysis including smoking status, alcohol consumption, tumor stage, surgical resection, induction chemotherapy, and total platelet count, the MPV remained a significant prognostic factor for CSS (HR 0.77, 95% CI 0.63–0.93, *p* = 0.008; Table 3).

**Table 2:** Univariate analyses of clinical-pathological parameters for the prediction of cancer-specific survival, locoregional control and recurrence-free survival

Patient characteristics	Cancer-specific survival		Locoregional control		Recurrence-free survival	
	HR (95% CI)	*p* value	HR (95% CI)	*p* value	HR (95% CI)	*p* value
Sex						
Male	1		1		1	
Female	1.24 (0.73–2.10)	0.437	1.08 (0.65–1.79)	0.775	1.01 (0.62–1.64)	0.959
Age at diagnosis						
< 60	1		1		1	
> 60	0.97 (0.60–1.59)	0.917	1.02 (0.65–1.59)	0.932	0.98 (0.64–1.49)	0.913
Smoking status						
Former or never	1		1		1	
Current	2.33 (1.31–4.14)	0.004	2.19 (1.30–3.68)	0.003	2.34 (1.43–3.82)	0.001
Alcohol consumption						
Former or never	1		1		1	
Current	1.73 (1.06–2.83)	0.029	1.90 (1.20–3.01)	0.006	1.88 (1.23–2.89)	0.004
Tumor grade						
G 1/2	1		1		1	
G 3/4	0.75 (0.46–1.22)	0.245	0.72 (0.46–1.12)	0.148	0.71 (0.47–1.08)	0.108
Tumor stage						
T 1/2	1		1		1	
T 3/4	3.53 (1.88–6.61)	< 0.001	4.99 (2.56–9.71)	< 0.001	3.62 (2.08–6.32)	< 0.001
Nodal involvement						
N0	1		1		1	
N+	1.55 (0.62–3.89)	0.345	1.21 (0.56–2.63)	0.632	1.42 (0.66–3.07)	0.375
Surgery						
Yes	1		1		1	
No	0.26 (0.14–0.46)	< 0.001	0.19 (0.11–0.35)	< 0.001	0.26 (0.16–0.42)	< 0.001
Induction chemotherapy						
Yes	1		1		1	
No	1.81 (1.05–3.10)	0.032	1.77 (1.08–2.88)	0.022	1.86 (1.18–2.94)	0.007
Concomitant chemotherapy						
Yes	1		1		1	
No	1.04 (0.61–1.77)	0.893	1.16 (0.69–1.93)	0.577	1.01 (0.64–1.61)	0.959
Platelet count (G/L)						
Median (mean ± SD)	1.004 (1.002–1.006)	< 0.001	1.004 (1.002–1.006)	< 0.001	1.003 (1.001–1.005)	0.001
MPV (fL)						
Median (mean ± SD)	0.85 (0.74–0.98)	0.025	0.86 (0.74–0.99)	0.034	0.87 (0.76–0.996)	0.043

MPV, mean platelet volume; HR, hazard ratio; CI, confidence interval; SD, standard deviation

**Table 3:** Multivariate analyses of clinical-pathological parameters for the prediction of cancer-specific survival, locoregional control and recurrence-free survival

	Cancer-specific survival*		Locoregional control*		Recurrence-free survival*	
	HR (95% CI)	*p* value	HR (95% CI)	*p* value	HR (95% CI)	*p* value
Smoking status						
Former/never	1		1		1	
Current	2.38 (1.25–4.53)	0.008	1.96 (1.09–3.50)	0.023	2.08 (1.21–3.60)	0.008
Alcohol consumption						
Former or never	1		1		1	
Current	0.99 (0.57–1.72)	0.973	1.06 (0.63–1.78)	0.818	1.12 (0.69–1.80)	0.65
Tumor stage						
T 1/2	1		1		1	
T 3/4	1.76 (0.79–3.92)	0.165	2.67 (1.13–6.28)	0.024	1.91 (0.93–3.89)	0.076
Surgery						
No	1		1		1	
Yes	0.25 (0.12–0.54)	< 0.001	0.26 (0.13–0.53)	< 0.001	0.33 (0.17–0.62)	0.001
Induction chemotherapy						
No	1		1		1	
Yes	0.79 (0.43–1.43)	0.429	0.80 (0.47–1.37)	0.428	0.92 (0.56–1.52)	0.753
Platelet count (continuous)	1.001 (0.999–1.004)	0.304	1.002 (1.000–1.004)	0.121	1.002 (0.999–1.004)	0.174
MPV (continuous)	0.77 (0.63–0.93)	0.008	0.80 (0.65–0.98)	0.030	0.83 (0.685–0.999)	0.049

MPV, mean platelet volume; HR, hazard ratio; CI, confidence interval

^*^Adjustment for all factors significantly associated in univariate analysis

In the analysis of LC, the MPV was significantly associated with LC in univariate analysis (HR 0.86, 95% CI 0.74–0.99, *p* = 0.034; Table 2) that also showed a significant relationship between smoking status, alcohol consumption, tumor stage, surgical resection, induction chemotherapy, and total platelet count and LC. In multivariate analysis that included parameters significantly associated with LC in univariate analysis, the MPV remained significantly associated with LC (HR 0.80, 95% CI 0.65–0.98, *p* = 0.030; Table 3).

Furthermore, univariate and multivariate analyses showed a significant association between the MPV and RFS (HR 0.87, 95% CI 0.76–0.996; *p* = 0.043 and HR 0.83, 95% CI 0.685–0.999, *p* = 0.049; Tables 2 and 3). Multivariate analysis also revealed a significant association between smoking and LC (HR 1.96, 95% CI 1.09–3.50, *p* = 0.23), RFS ( HR 2.08, 95% CI 1.21–3.60, *p* = 0.008), and CSS (HR 2.38, 95% CI 1.25–4.53, *p* = 0.008). Additionally, multivariate analysis identified the tumor stage as a significant predictor of LC (HR 2.67, 95% CI 1.13–6.28, *p* = 0.024), and surgical resection as a prognostic factor for LC (HR 0.26, 95% CI 0.13–0.53, *p* < 0.001), RFS (HR 0.33, 95% CI 0.17–0.62, *p* = 0.001), and CSS (HR 0.25, 95% CI 0.12–0.54, *p* < 0.001).

Among patients treated with definitive radio (chemo-) therapy, the pre-treatment MPV was identified as significant parameter for CSS (HR 0.68, 95% CI 0.50–0.91, *p* = 0.011), LC (HR 0.77, 95% CI 0.59–0.99, *p* = 0.045), and PFS (HR 0.77, 95% CI 0.60–0.99, *p* = 0.044; Table 4). In multivariate analysis, the MPV remained a significant predictor of CSS (HR 0.69, 95% CI 0.51–0.93, *p* = 0.014), in addition, a trend for an association of the MPV with LC (HR 0.80, 95% CI 0.62–1.04, *p* = 0.093) and PFS (HR 0.80, 95% CI 0.627–1.03, *p* = 0.085) was detected.

**Table 4:** Univariate analyses of clinical-pathological parameters for the prediction of cancer-specific survival, locoregional control and recurrence-free survival in patients treated with definitive radio (chemo/immuno-) therapy

Patient characteristics	Cancer-specific survival		Locoregional control		Recurrence-free survival	
	HR (95% CI)	*p* value	HR (95% CI)	*p* value	HR (95% CI)	*p* value
Sex						
Male	1		1		1	
Female	1.30 (0.69–2.45)	0.410	1.14 (0.64–2.04)	0.656	1.11 (0.63–1.94)	0.723
Age at diagnosis						
< 60	1		1		1	
> 60	0.76 (0.44–1.32)	0.325	0.78(0.48–1.28)	0.327	0.72 (0.45–1.15)	0.169
Smoking status						
Former or never	1		1		1	
Current	2.36 (1.21–4.61)	0.012	2.11 (1.19–3.73)	0.010	2.18 (1.26–3.78)	0.005
Alcohol consumption						
Former or never	1		1		1	
Current	1.72 (0.98–3.02)	0.061	1.65 (0.99–2.74)	0.052	1.59 (0.98–2.58)	0.062
Tumor grade						
G 1/2	1		1		1	
G 3/4	0.87 (0.49–1.53)	0.635	0.87 (0.53–1.42)	0.577	0.82 (0.51–1.32)	0.406
Tumor stage						
T 1/2	1		1		1	
T 3/4	1.79 (0.65–4.98)	0.262	2.273(0.83–6.26)	0.112	1.57 (0.68–3.64)	0.288
Nodal involvement						
N0	1		1		1	
N+	1.75 (0.63–4.87)	0.287	1.54 (0.66–3.59))	0.313	1.72 (0.74–3.99)	0.205
Induction chemotherapy						
Yes	1		1		1	
No	0.80 (0.44–1.45)	0.461	0.79 (0.47–1.34)	0.382	0.93 (0.57–1.53)	0.787
Concomitant chemotherapy						
Yes	1		1		1	
No	0.63 (0.29–1.34)	0.232	0.72 (0.35–1.45)	0.357	0.68 (0.35–1.34)	0.266
Chemo-/Immunotherapy						
No	1		1		1	
Erbitux	1.14 (0.48–2.68)	0.772	1.02 (0.45–2.32)	0.960	0.97 (0.44–2.13)	0.944
Cisplatin	0.51 (0.23–1.11)	0.091	0.64 (0.31–1.32)	0.228	0.61 (0.35–1.22)	0.160
RTx technique						
3D conformal	1		1		1	
IMRT	0.75 (0.42–1.33)	0.322	0.89 (0.53–1.49)	0.663	0.93 (0.57–1.53)	0.785
RTx total dose (Gy)						
≤ 60	1		1		1	
> 60 to < 70	1.51 (0.29–7.90)	0.625	1.57 (0.30–8.19)	0.593	2.69 (0.64–11.43)	0.178
≥ 70	0.51 (0.20–1.30)	0.160	0.61(0.24–1.53)	0.294	0.66 (0.26–1.65)	0.373
Platelet count (G/L)						
Median (mean ± SD)	1.003 (1.000–1.006)	0.025	1.004 (1.001–1.006)	0.004	1.003 (1.001–1.006)	0.007
MPV (fL)						
Median (mean ± SD)	0.68 (0.50–0.91)	0.011	0.77 (0.59–0.99)	0.045	0.77(0.60–0.99)	0.044

MPV, mean platelet volume; HR, hazard ratio; CI, confidence interval; SD, standard deviation; IMRT, intensity modulated radiation therapy

In the subgroup of patients treated with postoperative radio (chemo-) therapy, the pre-treatment MPV was not significantly associated with CSS, LC, and PFS (Table 5).

**Table 5:** Univariate analyses of clinical-pathological parameters for the prediction of cancer-specific survival, locoregional control and recurrence-free survival in patients treated with postoperative radio (chemo/immuno-) therapy

Patient characteristics	Cancer-specific survival		Locoregional control		Recurrence-free survival	
	HR (95% CI)	*p* value	HR (95% CI)	*p* value	HR (95% CI)	*p* value
Sex						
Male	1		1		1	
Female	1.49 (0.54–4.14)	0.445	1.50 (0.50–4.47)	0.471	1.15 (0.44–2.99)	0.778
Age at diagnosis						
< 60	1		1		1	
> 60	1.29 (0.45–3.68)	0.633	1.28 (0.44–3.70)	0.648	1.45 (0.59–3.51)	0.417
Smoking status						
Former or never	1		1		1	
Current	1.78 (0.56–5.64)	0.327	2.38 (0.66–8.68)	0.187	2.82 (0.94–8.50)	0.066
Alcohol consumption						
Former or never	1		1		1	
Current	0.77 (0.236–2.496)	0.661	1.52 (0.49–4.64)	0.468	1.76 (0.71–4.44)	0.220
Tumor grade						
G 1/2	1		1		1	
G 3/4	1.19 (0.383–3.723)	0.759	1.61 (0.45–5.79)	0.468	1.33 (0.48–3.67)	0.583
Tumor stage						
T 1/2	1		1		1	
T 3/4	2.48 (0.924–6.644)	0.071	3.20 (1.10–9.31)	0.033	2.85 (1.17–6.89)	0.021
Nodal involvement						
N0	1		1		1	
N+	2.67 (0.33–21.89)	0.359	1.29 (0.16–10.25)	0.808	1.96 (0.26–15.11)	0.517
Induction chemotherapy						
Yes	1		1		1	
No	0.10 (0.03–0.39)	0.001	9.19 (2.51–33.69)	0.001	5.90 (1.69–20.49)	0.005
Concomitant chemotherapy						
Yes	1		1		1	
No	0.36 (0.11–1.16)	0.086	0.24 (0.07–0.87)	0.030	0.29 (0.10–0.79)	0.016
Chemo-/Immunotherapy						
No	1		1		1	
Erbitux	n.a	0.984	n.a	0.983	n.a	0.98
Cisplatin	0.37 (0.12–1.19)	0.095	0.25 (0.07–0.90)	0.035	0.29 (0.11–0.82	0.019
RTx technique						
3D conformal	1		1		1	
IMRT	1.34 (0.40–4.45)	0.634	0.70 (0.21–2.33)	0.562	0.76 (0.28–2.07)	0.595
RTx total dose						
≤ 60	1		1		1	
> 60 to < 70	1.32 (0.40–4.34)	0.652	3.28 (0.72–14.98)	0.125	0.99 (0.38–2.56)	0.984
≥ 70	1.77 (0.39–8.09)	0.461	2.93 (0.41–20.79)	0.283	0.80 (0.17–3.87)	0.783
Platelet count (G/L)						
Median (mean ± SD)	1.002 (0.997–1.007)	0.404	1.002 (0.99–1.007)	0.535	1.002 (0.99–1.006)	0.516
MPV (fL)						
Median (mean ± SD)	0.87 (0.67–01.11)	0.257	0.83 (0.65–1.06)	0.128	0.87 (0.69–1.09)	0.239

MPV, mean platelet volume; HR, hazard ratio; CI, confidence interval; SD, standard deviation; IMRT, intensity modulated radiation therapy

**Fig. 1:**
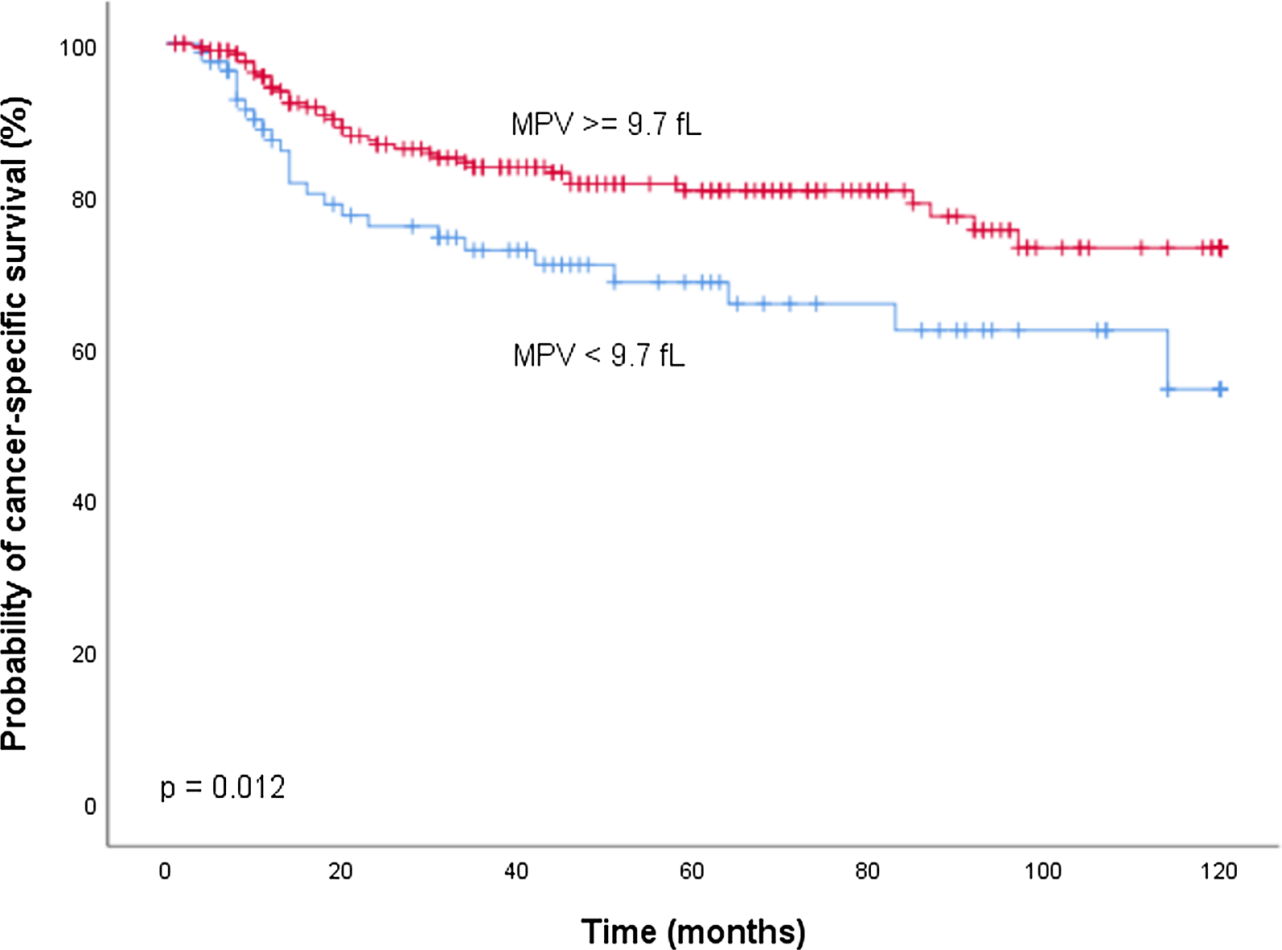
Kaplan Meier curves for cancer-specific survival by mean platelet volume (MPV)

**Fig. 2:**
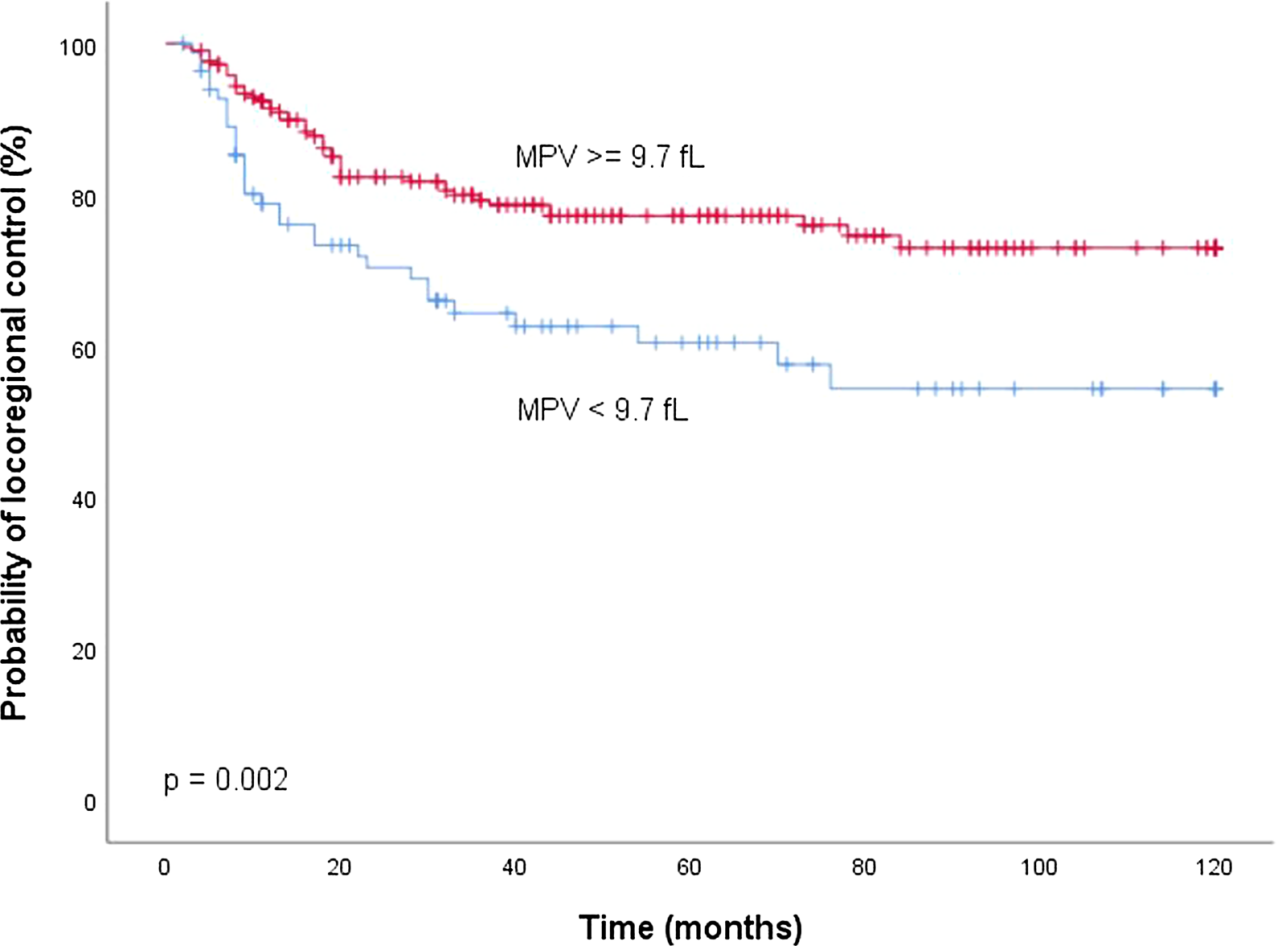
Kaplan Meier curves for locoregional control by mean platelet volume (MPV)

**Fig. 3:**
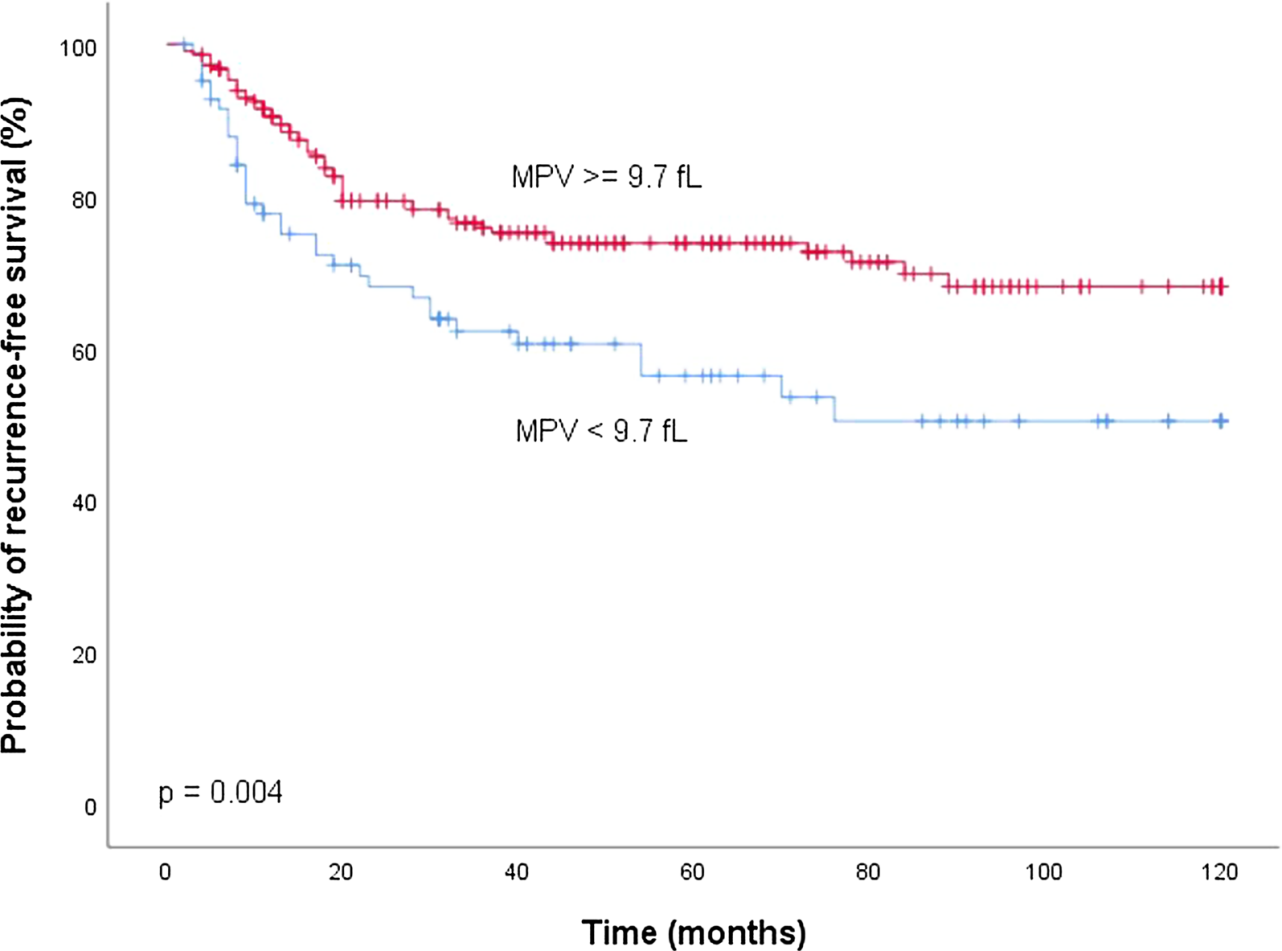
Kaplan Meier curves for recurrence-free survival by mean platelet volume (MPV)

To differentiate between a low and high MPV, an empiric cut-off at the 25th percentile of its distribution (9.7 fl) was used. Overall, there were 90 patients (29.2%) with a low MPV (< 9.7 fL) and 229 patients (71.8%) with a high MPV (≥ 9.7 fL). Kaplan Meier analysis demonstrated a significantly decreased CSS (*p* = 0.012, Fig. 1), RFS (*p* = 0.004, Fig. 2) and LC (*p* = 0.002, Fig. 3) for patients with a low MPV.

In a subgroup of 60 patients (19%), information on HPV status was available. In patients with HPV negative tumors (n = 23), the median MPV was 10.0 fL (mean 9.94 ± 1.15 fL) and in patients with HPV positive tumors (n = 37), the median MPV was 10.5 fL (mean 10.43 ± 0.90 fL, *p* = 0.001), respectively.

The analysis of the prognostic role of the MPV in patients with HPV negative tumors showed a significant relationship of the pre-treatment MPV with LC (HR 0.53, 95% CI 0.34–0.87, *p* = 0.012) and RFS (HR 0.58, 95% CI 0.36–0.93, *p* = 0.024) but not with CSS (HR 0.58, 95% CI 0.31–1.11, *p* = 0.102). In patients with HPV positive tumors, an association of the MPV with LC, RFS as well as CSS was not found (all *p* > 0.05).

## Discussion

In the present study, we analyzed the prognostic significance of the pre-treatment MPV in patients with OPSCC and detected a significant association between a decreased MPV and poor CSS, LC, and RFS.

Similar to our findings, previous studies have demonstrated that a low MPV is associated with poor outcome in patients with different solid tumors [13–18]. For instance, a recent study found a highly significant association of decreased MPV with RFS as well as with CSS in a large cohort of patients with non-metastatic renal cell carcinoma [17]. Furthermore, significant associations between low MPV values and poor prognosis have been reported in patients with hepatocellular, bladder cancers. In invasive bladder cancer patients decreased MPV was a negative predictor for OS (HR 2.023, 95% CI 1.050–3.897, *p* = 0.025), in lung cancers, it was a negative parameter for disease-free survival (HR 1.713; 95% CI 1.070–2.742, *p* = 0.025) and OS (HR 2.835; 95% CI 1.304–6.163, *p* = 0.009) [11, 12, 16]. A similar effect size has been detected concerning the relationship between the MPV and CSS in our study. However, different endpoints and potential confounders evaluated in previous studies make it difficult to compare these data with our results.

Data on the role of the association between MPV and prognosis in HNSCC patients are very limited. Park and colleagues aimed to establish a scoring system for patients with oral squamous cell carcinoma using platelet and MPV levels measured postoperatively and to evaluate their significance as prognostic factors [20]. The authors detected a significant association between the combination of a platelet (COP)-MPV score and survival and concluded that the COP-MPV score could be a prognostic factor in patients with oral cancer. However, the study only included a total of 40 patients. In a subsequent study on 115 head and neck cancer patients, a significant relationship between the COP-MPV score and prognosis was not detected [21].

In the present study, we observed that the pre-treatment MPV was an independent prognostic factor for outcome in OPSCC patients. To the best of our knowledge, we are the first to describe these results in a European cohort of patients with OPSCC. The major strength of our study is the relatively large cohort including 319 patients that represents, to our knowledge, the largest study population investigating the association between the MPV and prognosis in head and neck cancer patients. Furthermore, we have defined CSS as primary endpoint that directly reflects cancer prognosis and have identified a decreased MPV as an independent predictor of CSS. Another strength of our study is the relatively long follow-up period.

There is a convincing body of data demonstrating that patients with HNSCC who continue tobacco smoking have lower rates of complete response to radiation therapy and poorer survival, compared to nonsmokers and those who quit prior to treatment [23, 24]. Likewise, several data report the association between alcohol consumption and a decreased survival of patients with cancer oral cavity, pharynx and larynx [25]. According to the National Comprehensive Cancer Networt guidelines, tumor stage is an important prognostic factor for patients with HNSCC [26]. In our study, these parameters affected survival outcome in univariate analysis. In multivariate analysis, we identified current tobacco smoking and surgical resection as significant predictors of improved LC, RFS, and CSS, additionally, large tumor stage was associated with unfavorable LC rates. Our results suggest that the MPV might provide additional prognostic information besides these clinical characteristics and contribute to a better risk stratification and an improvement in oncological therapy decisions.

Recent experimental and clinical data indicate that the activation of platelets is crucial for cancer progression by promoting angiogenesis, degradation of the extracellular matrix, and release of adhesion molecules and growth factors [27, 28]. A number of platelet- expressed proteins have been demonstrated to be critical for metastatic dissemination in experimental animal models, in particular, metalloproteinase-9 (MMP-9) that has been shown to promote invasiveness of tumor cells [29] and beta-3 integrins that have been implicated to trigger bone metastasis formation [30]. Furthermore, circulating tumor cells encounter platelets and may activate them, resulting in the formation of microparticles that have been found to promote invasiveness of tumor cells [31]. Platelets are also involved in processes driving tumor angiogenesis, through the release of VEGF and other pro-angiogenic factors [7, 32]. In addition, various tumor-related humoral factors and pro-inflammatory cytokines such as interleukin (IL)-1, IL-3 and IL-6 have been shown to stimulate thrombopoiesis in cancer patients [33].

Our data support the hypothesis that a low MPV level is a prognostic factor for poor outcome in OPSCC patients. The main limitation of this study is the retrospective nature with all its possible shortcomings such as the potential impact of unmeasured confounders. In view of the lack of a standardized cut-off value, MPV was dichotomized into a binary variable with an arbitrary cut-off at the 25th percentile of its distribution. We used the cutoff mainly to provide a better visualization of the association between MPV and clinical outcome whereas a more precise measurement of this association is given by the hazard ratios and 95% confidence intervals. However, future investigations are necessary for the determination and validation of an optimal cut-off level. Furthermore, information on HPV status was available in only 60 patients. Nevertheless, we performed a subgroup analysis to separately evaluate the prognostic role of the MPV in patients with HPV negative and positive tumors and found an association between MPV and outcome in patients with HPV negative cancer but not in patients with HPV positive cancer that represents a distinct clinical and biologic entity with many unresolved issues. The explanation for this finding remains therefore speculative and should be investigated in future research.

## Conclusions

Our study shows that the decreased pre-treatment MPV is a prognostic factor for poor outcome in OPSCC patients. Nevertheless, validation of our findings in prospective studies is imperative to draw firm conclusions about the role of the MPV for OPSCC prognosis. If confirmed by additional studies, determination of the MPV might contribute to a better risk stratification and improved oncological therapy decisions in patients with OPSCC.

## Data Availability

The datasets used and/or analysed during the current study are available from the corresponding author on reasonable request.

## Abbreviations

MPV: Mean platelet volume
OPSCC: Oropharyngeal squamous cell cancer
CSS: Cancer-specific survival
LC: Locoregional control
RFS: Recurrence-free survival
HR: Hazard ratio
VEGF: Vascular endothelial growth factor
HNSCC: Head and neck squamous cell cancer
COP: Combination of a platelet
TPF: Docetaxel, cisplatin, 5-fluorouracil
CI: Confidence interval
HPV: Human papillomavirus
MMP-9: Metalloproteinase-9
IL: Interleukin
